# Analysis of Gender Differences in the Rotational Alignment of the Distal Femur in Kinematically Aligned and Mechanically Aligned Total Knee Arthroplasty

**DOI:** 10.3390/jcm10163691

**Published:** 2021-08-20

**Authors:** Byung-Woo Cho, Hyoung-Taek Hong, Yong-Gon Koh, Jeehoon Choi, Kwan-Kyu Park, Kyoung-Tak Kang

**Affiliations:** 1 Department of Orthopaedic Surgery, Severance Hospital, Yonsei University College of Medicine, 50-1 Yonsei-ro, Seodaemun-gu, Seoul 03722, Korea; chobw0704@yuhs.ac (B.-W.C.); ghoonie@yuhs.ac (J.C.); 2 Department of Mechanical Engineering, Yonsei University, 50 Yonsei-ro, Seodaemun-gu, Seoul 03722, Korea; hyoungtaekhong@gmail.com; 3 Joint Reconstruction Center, Department of Orthopaedic Surgery, Yonsei Sarang Hospital, 10 Hyoryeong-ro, Seocho-gu, Seoul 06698, Korea; osygkoh@gmail.com

**Keywords:** mechanically aligned total knee arthroplasty, kinematically aligned total knee arthroplasty, rotational alignments

## Abstract

To compare the angle between the external rotation references of the femoral components in the axial plane by gender and lower limb alignment in Korean patients with osteoarthritis (OA). Magnetic resonance (MR) images of 1273 patients were imported into a modeling software and segmented to develop three-dimensional femoral bony and cartilaginous models. The surgical transepicondylar axis (sTEA), posterior condylar axis (PCA), the kinematically aligned axis (KAA), and anteroposterior axis were used as rotational references in the axial plane for mechanically aligned (MA) TKA. The relationship among axes were investigated. Among 1273 patients, 942 were female and 331 were male. According to lower limb alignment, the varus and valgus knee groups comprised 848 and 425 patients, respectively. All measurements, except PCA-sTEA, differed significantly between men and women; all measurements, except PCA-sTEA, did not differ significantly between the varus and valgus knee groups. In elderly Korean patients with OA, rotational alignment of the distal femur showed gender differences, but no differences were seen according to lower limb alignment. The concern for malrotation of femoral components during kinematically aligned TKA is less in Koreans than in Caucasians and relatively less in women than in men. In MA TKA, malrotation of the femoral components can be avoided by setting different rotational alignments for the genders.

## 1. Introduction

Proper rotational alignment of the prosthesis is essential for successful outcomes in total knee arthroplasty (TKA). Since external rotation of the femoral component affects patellar tracking, patellofemoral joint contact, varus-valgus stability during knee flexion, and rotational alignment of tibial components [1,2,3], it is important to find an optimal rotational reference.

The measured resection technique of mechanically aligned (MA) TKA is a conventional method wherein the rotational alignment of the femoral component is determined using bony landmarks. The main landmarks used are the surgical transepicondylar axis (sTEA), posterior condylar axis (PCA), and anteroposterior axis (APA), but it is not yet clear which one is superior to the others [4]. In kinematically aligned (KA) TKA, which is reported to have similar or better outcomes than MA TKA [5], the amount of bone resection of the distal and posterior femoral condyles with adjustments for cartilage wear is the same as the condylar thickness of the femoral component [6]. Park et al. compared the flexion-extension axis (FEA) of the tibia formed in KA TKA with the axes used in MA TKA in the axial plane and reported that the FEA in KA TKA is 4° internally rotated compared to the sTEA [7].

Many studies have reported rotational alignment of the distal femur in the axial plane, with varied results [7,8,9,10,11,12,13,14,15,16,17]. The reason for this discrepancy is that the test modalities were not the same for each study [18,19], and each cohort had different characteristics. The shape and size of the distal femur show gender-related differences in both Asians and Caucasians [11,20,21,22,23,24]; therefore, it can be inferred that there will be a difference in the relationship between the axes. Moreover, since valgus knees show hypoplasia of the lateral condyle compared to varus knees [25], changes can be induced in the reference angles. In addition, since it is difficult to identify bony landmarks in the knees of elderly people or patients with severe osteoarthritis (OA) [26], cohort selection also contributes to a significant difference.

Therefore, the aim of this study was to compare the angle between the external rotation references of the femoral components in the axial plane according to gender and lower limb alignment using a large dataset of Korean patients with OA. We hypothesized the following: (1) there are differences in the reference angles used in MA TKA and KA TKA according to gender; and (2) there are differences in the reference angles used in MA and KA TKA according to the alignment of the lower limbs.

## 2. Materials and Methods

### 2.1. Patient Recruitment

Institutional review board approval was obtained for this study. A total of 1273 consecutive patients who underwent TKA at our institution underwent magnetic resonance (MR) imaging. The average age of the patients was 70.08 ± 6.66 years, and all subjects had OA without rheumatoid arthritis. The exclusion criteria were any history of osteotomy or fracture of the affected limb.

### 2.2. Three-Dimensional Model Reconstruction

MR images were obtained according to a standard protocol using a 1.5-T MR imaging scanner (Achieva 1.5 T; Philips Healthcare, Best, The Netherlands) in patients with end-stage OA waiting for TKA. Images in the sagittal plane were obtained using 1-mm high-resolution slice thickness for the tibiofemoral knee joint and 5-mm slice thickness for the hip and ankle joints in the axial plane. Under non-fat saturation conditions, the MR images consisted of axial proton density sequences. A high-resolution setting was used for the spectral pre-saturation inversion recovery sequence (echo time, 25.0 ms; repetition time, 3590.8 ms; acquisition matrix, 512 × 512 pixels; number of excitations, 2.0; field of view, 140 × 140 mm). MR images were imported into a modeling software (Mimics version 17.0; Materialize, Leuven, Belgium) and segmented to develop three-dimensional (3D) femoral bony and cartilaginous models.

The mechanical axes of the femur and tibia were visualized using the 3D reconstructed model. The mechanical axis of the femur was defined as the line between the center of the hip and the intercondylar notch. The mechanical axis of the tibia was defined as the line between the center of the proximal tibia and the center of the ankle. A varus knee was defined if the medial hip–knee–ankle angle was ≤180°, and a valgus knee was defined if the medial angle was >180°. The plane perpendicular to the femoral mechanical axis was defined as the axial plane. The sTEA was projected on the axial plane, and the projected line was used as the x-axis. The coronal plane was defined using the mechanical axis and x-axis. The sagittal plane was defined as the plane perpendicular to the axial and coronal planes.

### 2.3. Measurement Methods

**Figure 1:**
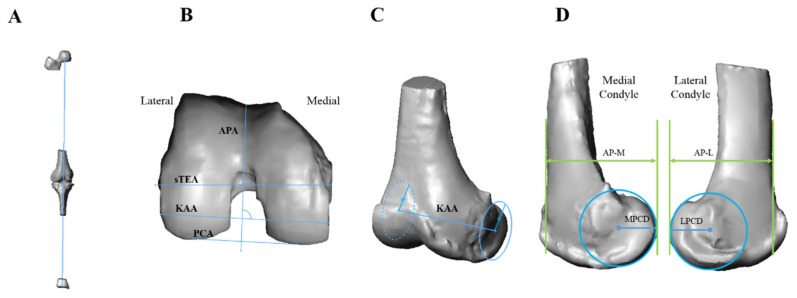
Definition of measurements. (**A**) Hip–knee–ankle angle was defined as the medial angle between mechanical axes of femur and tibia. (**B**) Rotational references of axial plane in mechanically aligned total knee arthroplasty (TKA), anteroposterior axis (APA), surgical transepicondylar axis (sTEA), posterior condylar axis (PCA), and kinematically aligned axis (KAA). (**C**) Definition of KAA. The best-fit circle to non-affected posterior femoral condyle was propagated to the affected condyle and was located to best fit. The diameters of the two circles in this composite are the same. (**D**) Medial posterior condylar diameter (MPCD) and lateral posterior condylar diameter (LPCD) were defined as the radii of the circles, best fit to each posterior femoral condyle. MPCD and LPCD are not the same.

The sTEA, PCA, and APA were used as rotational references in the axial plane for MA TKA (Figure 1B). The sTEA was defined as a line connecting the most prominent lateral epicondyle and the medial epicondylar sulcus [27]. The PCA was defined as a line tangential to the most prominent point of the posterior condyle [28]. The APA was defined as the line connecting the deepest point on the trochlear groove and the intercondylar notch [28]. As described by Howell et al., KA TKA uses a 9-mm thick implant with a symmetric femoral condyle [6]. The FEA of the tibiofemoral joint formed after KA TKA was defined as in the study by Park et al. [7]. A circle was drawn to fit the distal and posterior cartilaginous contours of a non-affected condyle without cartilaginous defects on the sagittal MR image. This circle was propagated to the affected condyle and was best fit with the cartilaginous contour. If there was cartilage wear in the affected condyle, the circle was placed by assuming an imaginary contour with a cartilage thickness of 2 mm [29]. The line connecting the centers of these two circles was projected onto the axial plane and was defined as the FEA of the tibiofemoral joint formed after KA TKA, that is, the kinematically aligned axis (KAA) (Figure 1C). The axes used in MA TKA and the KAA were compared. If the value of A-B was positive, B was externally rotated in relation to A.

In the sagittal MR images, the anteroposterior length (AP) of the distal femur was defined as the distance between the anterior border of the femoral shaft cortex and a line tangential to the posterior femoral condyle, and was divided into AP-M (medial condyle) and AP-L (lateral condyle). The diameters of the lateral and medial posterior condyles were defined as the radii of the circles, best fit to each posterior femoral condyle (Figure 1D).

### 2.4. Statistical Analysis

Statistical analyses were performed using SPSS software for Windows (version 12.0; SPSS, Chicago, IL, USA). The Student’s *t*-test was performed to determine the significance of any gender differences. Probability (*p*) values < 0.05 were considered statistically significant. A power analysis was performed to exclude overpowering due to the large number of cases. A post hoc power analysis of the study for detecting differences in each angle between the genders was performed using G power 3.1 (Heinrich Heine Universität Düsseldorf, DE. One-tailed analysis with an alpha value of 0.05 was performed. The power analysis determined that 1237 patients (942, female and 331, male) represented a sufficient size, as our power values for each angle, except the sTEA-PCA angle, were >80.0%.

## 3. Results

Among the 1273 patients, 942 were female and 331 were male. According to lower limb alignment, the varus and valgus knee groups comprised 848 and 425 patients, respectively.

There were statistical differences between men and women in all measurements, except the PCA-sTEA values (Table 1). The angular differences between the KAA and the axes used in MA TKA for each gender showed a disparity of >4.5°, but the angular differences among the axes used in MA TKA showed a disparity of <2°. Compared to the sTEA, the KAA was 4.02° ± 3.38° externally rotated in women and 0.56° ± 4.11° internally rotated in men; compared to the PCA, the KAA was 6.50° ± 3.64° externally rotated in women and 1.78° ± 4.20° externally rotated in men; and compared to the APA, the KAA was 92.66° ± 1.94° externally rotated in women and 86.65° ± 6.61° externally rotated in men. Compared to the sTEA, the APA was 91.36° ± 2.79° externally rotated in women and 92.79° ± 4.51° externally rotated in men; compared to the PCA, the APA was 93.84° ± 2.93° externally rotated in women and 95.13° ± 4.67° externally rotated in men. The PCA-sTEA value had a statistical power of 0.426, which is not reliable. The absolute value and the ratio of the posterior condylar diameter to the AP were significantly higher in men.

There were no statistical differences in all measurements, except for PCA-sTEA values, between the varus and valgus knee groups (Table 2). The PCA-sTEA value had a statistical power of 0.173, which is not reliable. The absolute value and the ratio of the posterior condylar diameter to the AP did not show any difference according to lower limb alignment.

The differences between the medial and lateral posterior condylar diameters were <0.2 mm in all cases irrespective of gender and lower limb alignment; no statistical difference was noted (Table 3).

## 4. Discussion

The most important finding of this study was that in a large dataset of elderly Korean patients with OA, the differences between the FEA of the tibiofemoral joint formed after KA TKA and the axes used in MA TKA, with the exception of PCA-sTEA, showed a disparity according to gender. These results satisfied our hypothesis. In the comparison between varus and valgus knee groups, there was no difference, except for that in the PCA-sTEA, which did not satisfy our hypothesis.

Prior to the interpretation of the results, the KAA used in this study was considered. The KAA is the theoretical FEA formed after KA TKA using a single radius implant and is different from the cylindrical axis (CA), which is considered to be the FEA of the preoperative natural knee. KA TKA aims to restore the kinematics of the pre-arthritic knee [30], and the FEA formed after surgery has been considered as a CA under the assumption that both condyles have a single radius of curvature [31,32]. However, the KAA is different from the CA due to the following reasons: first, unlike the lateral condyle, the medial condyle has double radii of curvature. Eckhoff et al. reported that the contour from 10° to 160° of the posterior condyle has a single radius of curvature, and the multi-radial curvature reported by other studies is an artifact that occurs when the actual knee is projected onto traditional orthogonal planes [32]. However, in the study by Blaha et al. using 65 pairs of cadaveric femurs [33] and in our study that avoided projection-induced distortion through 3D reconstruction, it was confirmed that some of the medial condyles have multiple radii of curvature. If an implant with a single radius of curvature is inserted [34] after distal and posterior bone resection of 9 mm in a medial condyle with double radii of curvature, subtle changes occur in the positions of the most distal and posterior points, and the articular contour between them. Unless it is a customized implant, these differences are inevitable, but the magnitude is not expected to be large [35]. In our study, it was assumed that KA TKA was performed using a single-radius implant to define the KAA. Therefore, when it was difficult to find the best-fit circle due to double radii of curvature, the circle drawn based on the contour compensated by the cartilage of another condyle was propagated and placed in contact with the tangents of the distal and posterior contour. Second, the radius of curvature of 0°–90° of the medial and lateral condyles is the same in most implants but differs in the actual knee [36]. Howell et al. reported that the radii of the medial and lateral femoral condyles in varus and valgus knees in patients with end-stage OA were not clinically important (≤0.2 mm) [37], and similar results were shown in our study. However, these minimal differences can induce an angular disparity in the axial plane. Taken together, the KAA defined in our study was the same as the FEA formed after KA TKA, but there may have been a slight difference from the CA of the natural knee before surgery.

We presented a comprehensive comparison of the axes used in KA TKA and MA TKA. The difference between men and women for the axes used in MA TKA can be utilized in actual surgery, but the KAA cannot be used as a reference in actual surgery. It is passively formed after KA TKA with equal resection of the distal and posterior condyles. Similar to those of the study of Park et al., our results regarding the KAA are meaningful in that we can predict the extent of femoral component rotation in KA TKA relative to the existing axes used in MA TKA in Asians. Although the study by Park et al. did not distinguish between men and women, the FEA formed after KA TKA was internally rotated by 4° compared to the sTEA, suggesting that there is concern for patellar maltracking and anterior knee pain [7]. Internal rotation of the femoral component causes patellofemoral complications [38,39] and can lead to poor clinical outcomes [40]. Therefore, there is a possibility of component malrotation in KA TKA targeting Caucasians. However, in our study of Koreans, the KAA showed 4.02° external rotation compared to the sTEA in women and 0.56° internal rotation compared to the sTEA in men; therefore, there is relatively less concern for malrotation of femoral components among Korean patients.

There are many differences in the basic anatomical structure, kinematics, and arthritic patterns between male and female knees. Since women’s knees show mediolaterally narrow and trapezoidal axial shapes [11,20,21,22] and the orientation of the trochlear groove is also different from that of men’s knees [41], there is a difference in orientation of the axes using bony landmarks. In addition, the sagittal curvature diameters [36] and posterior condylar offset [18,42], which are thought to have an effect on KAA, show a disparity between men and women. Therefore, if the same angular reference is applied in TKA, the anatomical diversity between men and women would be ignored.

The disparity between PCA and KAA was larger in women in our study. According to the definitions used in our study, the KAA was assumed to involve normal cartilage, and the PCA reflected actual cartilage defects. If there were no cartilage defects, the PCA and KAA were parallel. However, women kinematically receive more valgus momentum [43,44] and show valgus alignment and lateral compartment OA more frequently [45] than men. According to Nam et al., the rate of cartilage wear of the medial posterior condyle was 2% in varus knees, whereas the rate of wear of the lateral posterior condyle was 55% in valgus knees. [46]. Therefore, the prevalence of cartilage wear of the lateral posterior condyle is relatively higher in women, leading to a larger disparity between the PCA and KAA than that in men.

Since lateral condylar hypoplasia is common in valgus knees, we assumed that the value of the PCA-sTEA would be larger than that in varus knees. However, in reality, there was no statistical difference, and both values were <3°. In a study by Patel et al. using 557 MR imaging results, there were statistical differences between the varus (2.23° ± 1.57°) and valgus knee groups (2.88° ± 1.70°) [37]. There was a statistical difference between the varus (2.2° ± 1.0°) and valgus knee groups (3.0° ± 1.2°) in the study by Koh et al. using 1522 MR imaging results [11]. Our study using 1273 MR imaging scans showed similar results: 2.42° ± 1.49° and 2.49° ± 1.84° in the varus and valgus knee groups, respectively. Surgical instruments are generally set to an external rotation of 3°–5° based on the PCA, but our results suggest that these pre-set angles may be different from actual situations reflecting the cartilage.

As mentioned previously, the rotational alignments of the distal femur vary greatly depending on the study (Table 4). It seems that the results were affected by test modality [18,19] and number of cases, as well as ethnicity, age, sex, and the severity of OA. Even in studies comparing gender differences, the statistical significance appeared to be different, and this also seemed to be affected by the above variables (Table 5). From this point of view, the strengths of our study are as follows. First, this study was conducted only on Korean patients with OA who underwent TKA, and the number of cases was large enough. The large variability in femoral rotational alignment is well known, and in our study, all measurements showed a large range. Therefore, we set a sufficiently large sample size to remove the selection bias. In addition, statistical power was determined to exclude overpowering. Second, our study reflected the actual cartilage condition using MR imaging. According to an MR imaging study by Nam et al., it was reported that the thickness of the cartilage of the posterior femoral condyle was different between the medial and lateral compartments of Kellgren-Lawrence grade 3 and 4 knees [46]. Since the status of the cartilage of the posterior condyle affects the PCA, KAA, and CA, our MR imaging data reflect reality better than studies using computed tomography.

## 5. Conclusions

In elderly Korean patients with OA, the rotational alignment of the distal femur differed according to gender but not according to the alignment of the lower limb. When performing KA TKA in Koreans, the concern for malrotation of femoral components is less than that in Caucasians and relatively less in women than in men. In MA TKA, malrotation of the femoral components can be avoided by setting different rotational alignments for each gender.

## Figures and Tables

**Table 1:** Comparison of measurements according to gender.

Parameters	Whole Patients (*n* = 1273)	Female (*n* = 942)	Male (*n* = 331)	*p*-Value	Power
Age	70.08 ± 6.66 (41, 89)	70.32 ± 6.53 (41, 87)	69.39 ± 6.96 (49, 89)	n.s.	
sTEA-KAA (°)	2.73 ± 4.10 (−9.87, 14.81)	4.02 ± 3.38 (−9.87, 14.81)	−0.56 ± 4.11 (−7.1, 11.79)	<0.05	1.00
PCA-KAA (°)	5.18 ± 4.32 (−5.81,18.09)	6.50 ± 3.64 (−5.81, 18.09)	1.78 ± 4.20 (−3.9, 14.32)	<0.05	1.00
APA-KAA (°)	91.00 ± 4.59 (71.2, 105.9)	92.66 ± 1.94 (86.8, 98.4)	86.65 ± 6.61 (71.2, 105.9)	<0.05	1.00
sTEA-APA (°)	91.73 ± 3.38 (69.1, 105)	91.36 ± 2.79 (81.43, 101.53)	92.79 ± 4.51 (69.1, 105)	<0.05	1.00
PCA-APA (°)	94.18 ± 3.52 (71.9, 107.3)	93.84 ± 2.93 (81.45, 104.29)	95.13 ± 4.67 (71.9, 107.3)	<0.05	1.00
PCA-sTEA (°)	2.44 ± 1.62 (−8.95, 13.29)	2.48 ± 1.73 (−8.95, 13.29)	2.34 ± 1.23 (−8.23, 3.91)	<0.05	0.426
MPCD (mm)	40.62 ± 5.00 (24.76, 56.9)	39.17 ± 4.52 (24.76, 56.9)	44.73 ± 3.92 (35.6, 54.2)	<0.05	1.00
LPCD (mm)	40.55 ± 4.95 (25.67, 60.75)	39.01 ± 4.31 (25.67, 60.75)	44.92 ± 3.92 (34.33, 54.27)	<0.05	1.00
MPCD/AP	0.693 ± 0.080 (0.438, 1.032)	0.680 ± 0.079 (0.438, 1.032)	0.728 ± 0.071 (0.549, 0.964)	<0.05	1.00
LPCD/AP	0.692 ± 0.082 (0.452, 1.019)	0.677 ± 0.078 (0.452, 1.019)	0.731 ± 0.081 (0.542, 1.015)	<0.05	1.00

Values are presented as the mean ± standard deviation (range). sTEA, surgical transepicondylar axis; KAA, kinematically aligned axis; PCA, posterior condylar axis; APA, anteroposterior axis; MPCD, medial posterior condylar diameter; LPCD, lateral posterior condylar diameter; AP, anteriopesterior length; n.s., not significant.

**Table 2:** Comparison of measurements according to the lower limb alignment.

Parameters	Whole Patients (*n* = 1273)	Varus (*n* = 848)	Valgus (*n* = 425)	*p*-Value	Power
Age	70.08 ± 6.66 (41, 89)	70.19 ± 6.39 (49, 88)	69.85 ± 7.16 (41, 89)	n.s.	
sTEA-KAA (°)	2.87 ± 4.11 (−9.87, 14.81)	2.80 ± 4.11 (−9.87, 14.81)	3.01 ± 4.12 (−5.8, 14.52)	n.s.	
PCA-KAA (°)	5.31 ± 4.33 (−5.81,18.09)	5.22 ± 4.29 (−5.81,18.09)	5.48 ± 4.41 (−4.48,17.65)	n.s.	
APA-KAA (°)	89.01 ± 4.59 (74.1, 108.8)	88.83 ± 4.51 (74.1, 105.7)	89.05 ± 4.75 (79.8, 108.8)	n.s.	
sTEA-APA (°)	91.73 ± 3.38 (69.1, 105)	91.63 ± 3.37 (69.1, 105)	91.94 ± 3.41 (77.5, 104)	n.s.	
PCA-APA (°)	94.18 ± 3.52 (71.9, 107.3)	94.06 ± 3.49 (71.9, 105.7)	94.42 ± 3.56 (79.8, 107.3)	n.s.	
PCA-sTEA (°)	2.44 ± 1.62 (−8.95, 13.29)	2.42 ± 1.49 (−8.95, 13.18)	2.49 ± 1.84 (−7.15, 13.29)	<0.05	0.173
MPCD (mm)	40.62 ± 5.00 (24.76, 56.9)	40.67 ± 5.04 (24.76, 56.9)	40.51 ± 4.94 (29.17, 54.2)	n.s.	
LPCD (mm)	40.55 ± 4.95 (25.67, 60.75)	40.57 ± 5.02 (25.67, 60.75)	40.49 ± 4.81 (28.88, 53.4)	n.s.	
MPCD/AP	0.693 ± 0.080 (0.438, 1.032)	0.694 ± 0.079 (0.438, 0.964)	0.690 ± 0.081 (0.492, 1.032)	n.s.	
LPCD/AP	0.692 ± 0.082 (0.452, 1.019)	0.693 ± 0.084 (0.471, 1.019)	0.689 ± 0.078 (0.452, 0.993)	n.s.	

Values are presented as the mean ± standard deviation (range). sTEA, surgical transepicondylar axis; KAA, kinematically aligned axis; PCA, posterior condylar axis; APA, anteroposterior axis; MPCD, medial posterior condylar diameter; LPCD, lateral posterior condylar diameter; AP, anteriopesterior length; n.s., not significant.

**Table 3:** Comparison of the medial and lateral posterior condylar diameters according to gender and lower limb alignment.

Parameters	MPCD (mm)	LPCD (mm)	Deference (mm)	*p*-Value
Whole	40.62 ± 5.00	40.55 ± 4.95	0.07	0.304
Divided by gender				
Male	44.73 ± 3.92	44.92 ± 3.92	0.19	0.225
Female	39.17 ± 4.52	39.01 ± 4.31	0.16	0.303
Divided by lower limb alignment				
Varus	40.67 ± 5.04	40.57 ± 5.02	0.1	0.355
Valgus	40.51 ± 4.94	40.49 ± 4.81	0.02	0.473

Values are presented as the mean ± standard deviation.

**Table 4:** Comparison of angles between axial references with those in previous studies.

	Population	Cases	Female (%)	Age	Modality	sTEA-APA (°)	PCA-APA (°)	PCA-sTEA (°)	sTEA-KAA (°)	PCA-KAA (°)	APA-KAA (°)
Our study	Korea	1273	74.0	70.1	MRI	91.73 ± 3.38	94.18 ± 3.52	2.44 ± 1.62	2.73 ± 4.10	5.18 ± 4.32	91.00 ± 4.59
Koh et al. [11]	Korea	1522	85.3	68.9	MRI	91.2 ± 2.8	93.7 ± 3.0	2.2 ± 1.0			
Patel et al. [37]	USA	557	61.8	66.5	MRI	90.361 ±2.2		2.38 ± 1.62			
Park et al. [7]	USA	114	56.1	64	MRI	92.4 ± 3.5	96.9 ± 3.2	4.5 ± 2.4	−4.0 ± 2.5	0.5 ± 1.8	83.6 ± 3.1
Griffin et al. [8]	USA	104	60.6	42.8	MRI			3.11 ± 1.75			
Pun et al. [8]	India	40		29.5	MRI	92.7 ± 1.32		4.67 ±1.36			
Twiggs et al. [16]	Australia	726	57.0	69.1	CT	90.69 ± 5.26	92.54 ±5.11	1.85 ± 1.834			
Luyckx et al. [12]	Belgium	231	65.4	68.8	CT	94.8 ± 3.3		1.6 ± 1.9			
Jabalameli et al. [10]	Iran	108	60.2	65.2	CT		93.7 ± 2.1	1.6 ± 1.7			
Ng et al. [13]	Asian	50	38	64	CT	94.4 ± 3.5		1.9 ± 1.8			
Yip et al. [23]	China	41	17.1	78	Cadaver		97.0 ± 2.5				

**Table 5:** Comparison of gender differences in the angle between the axes used in mechanically aligned total knee arthroplasty with those in other studies.

	sTEA-APA (°)			PCA-APA (°)			PCA-sTEA (°)		
	Female	Male	*p*-Value	Female	Male	*p*-Value	Female	Male	*p*-Value
Our study	91.36 ± 2.79	92.79 ± 4.51	<0.05	93.84 ± 2.93	95.13 ± 4.67	<0.05	2.48 ± 1.73	2.34 ± 1.23	<0.05 ^†^
Koh et al. [11]	91.3 ± 2.8	90.7 ± 2.7	<0.05	93.8 ± 3.0	93.1 ± 2.7	<0.05	2.2 ± 1.1	2.0 ± 1.0	<0.05
Patel et al. [37]	90.245 ± 2.35	90.5484 ± 2.16	n.s				2.56 ± 1.59	2.08 ± 1.62	<0.05
Griffin et al. [8]							3.33 ± 1.82	2.75 ± 1.61	n.s.
Luyckx et al. [12]	94.6 ± 3.6	95.0 ± 3.3	n.s				1.7 ± 2.0	1.3 ± 1.8	n.s.
Berger et al. [47]							0.3 ± 1.2	3.5 ± 1.2	<0.05
Yip et al. [23]	92.36 ± 1.6	1.59 ± 2.8	n.s	98.24 ± 2.4	6.73 ± 2.5	n.s	5.8 ± 1.8	5.1 ± 1.9	<0.05

Values are presented as the mean ± standard deviation. sTEA, surgical transepicondylar axis; APA, anteroposterior axis; PCA, posterior condylar axis. ^†^ Statistical power = 0.426. n.s., not significant.

## Data Availability

The data presented in this study are available on request from the corresponding author. The data are not publicly available due to privacy.
